# The effect of fampridine on working memory: a randomized controlled trial based on a genome-guided repurposing approach

**DOI:** 10.1038/s41380-024-02820-1

**Published:** 2024-11-08

**Authors:** Andreas Papassotiropoulos, Virginie Freytag, Nathalie Schicktanz, Christiane Gerhards, Amanda Aerni, Tamás Faludi, Ehssan Amini, Elia Müggler, Annette Harings-Kaim, Thomas Schlitt, Dominique J.-F. de Quervain

**Affiliations:** 1 Division of Molecular Neuroscience, Department of Biomedicine, University of Basel, CH-4055 Basel, Switzerland; 2 Research Cluster Molecular and Cognitive Neurosciences, Department of Biomedicine, University of Basel, CH-4055 Basel, Switzerland; 3 Psychiatric University Clinics, University of Basel, CH-4055 Basel, Switzerland; 4 Division of Cognitive Neuroscience, Department of Biomedicine, University of Basel, CH-4055 Basel, Switzerland

**Keywords:** Psychiatric disorders, Drug discovery

## Abstract

Working memory (WM), a key component of cognitive functions, is often impaired in psychiatric disorders such as schizophrenia. Through a genome-guided drug repurposing approach, we identified fampridine, a potassium channel blocker used to improve walking in multiple sclerosis, as a candidate for modulating WM. In a subsequent double-blind, randomized, placebo-controlled, crossover trial in 43 healthy young adults (ClinicalTrials.gov, NCT04652557), we assessed fampridine’s impact on WM (3-back d-prime, primary outcome) after 3.5 days of repeated administration (10 mg twice daily). Independently of baseline cognitive performance, no significant main effect was observed (Wilcoxon *P* = 0.87, r = 0.026). However, lower baseline performance was associated with higher working memory performance after repeated intake of fampridine compared to placebo (r_s_ = −0.37, *P* = 0.014, *n* = 43). Additionally, repeated intake of fampridine lowered resting motor threshold (F(1,37) = 5.31, *P* = 0.027, R^2^β = 0.01), the non-behavioral secondary outcome, indicating increased cortical excitability linked to cognitive function. Fampridine’s capacity to enhance WM in low-performing individuals and to increase brain excitability points to its potential value for treating WM deficits.

## Introduction

Working memory (WM) represents a neural network with limited capacity capable of actively maintaining task-relevant information for a short time (i.e., seconds to minutes) [1, 2]. WM has a pivotal role in human cognition, allowing the integration of information from instantly perceived stimuli, long-term-memory and thought processes. WM deficits are key symptoms of psychiatric disorders, including schizophrenia [3–6], bipolar disorder [7], and attention-deficit hyperactivity disorder (ADHD) [8]. Moreover, WM is an important endophenotype in neuropsychiatric research and its use in human genetic studies has increased our understanding of mental disease [9–14]. In schizophrenia, strong and extensive evidence identifies WM as a key endophenotype. WM and WM-related neurofunctional traits are impaired in patients with schizophrenia and in their unaffected relatives [12, 15–18]. Importantly, WM deficits precede the onset of disease. Children who are predisposed to developing schizophrenia in adulthood experience increasing WM deficits as they age [19]. Heritability estimates of WM are substantial in nonclinical samples and comparable in schizophrenia [20–23]. Research on WM yields a common consensus that it constitutes a complex trait and that the buffer size for the transiently stored content varies inter-individually, which is partly due to genetic contribution [24]. Notably, genetic factors associated with WM and WM-related neurofunctional traits are also associated with the risk for schizophrenia [14, 25–28]. Thus, improving our understanding of the molecular basis of WM is predicted to inform future drug discovery and better treatment options in psychiatry [29].

Advances in the development of high-throughput genotyping platforms, analytical software, and large collaborative efforts have led to the identification of numerous well-validated genetic risk factors for common, complex diseases (http://www.genome.gov/gwastudies). Recent large-scale studies have also led to the robust identification of common and rare genetic risk factors for psychiatric disorders (for example [30–32]) and to the notion that many of these factors are shared across diagnostic categories [33, 34]. Large investments have been made with the expectation that genome-wide association study (GWAS) findings will ultimately lead to novel therapeutic agents. Important evidence came from a study which assessed the utility of GWAS in identifying alternative indications of existing drugs [35]. This study demonstrated that genes identified through significant GWAS hits are significantly more likely to be druggable or biopharmable targets than expected by chance. Importantly, the study showed that GWAS data may lead to immediate translational opportunities for drug discovery and development through successful drug repurposing [35]. Subsequent work, aimed at addressing the efficiency and innovation problem in drug development, has proposed approaches to catalyze the use of genomic information to support drug target validation, match target to disease indications, and identify rational repurposing opportunities for licensed drugs [36].

Given the above information it is reasonable to assume that GWAS have the potential of translating also into novel treatment targets for psychiatric conditions. Indeed, strategies for applying human genetics to drug discovery in clinical neuroscience [37] have already led to the identification of promising repurposing candidates [38–40] and to the initiation of clinical trials (for example ref. [41]). Notwithstanding this exciting progress, it is important to point out that the impact of GWAS-based research on drug discovery stands and falls with the ability to link the reported genetic associations to tractable and druggable mechanisms [37, 42]. Such mechanisms should be intrinsically involved in the disease under study and, ideally, should point to druggable disease targets. These considerations are particularly pertinent to the genetic study of mental disorders. The sole use of general psychiatric diagnoses as the trait of interest does not readily establish a link to objective, tractable, and druggable correlates of underlying mechanisms. To leverage the therapeutic significance of GWAS-supported findings, it is important to add and integrate information about relevant cognitive/emotional neuropsychiatric domains.

**Fig. 1:**
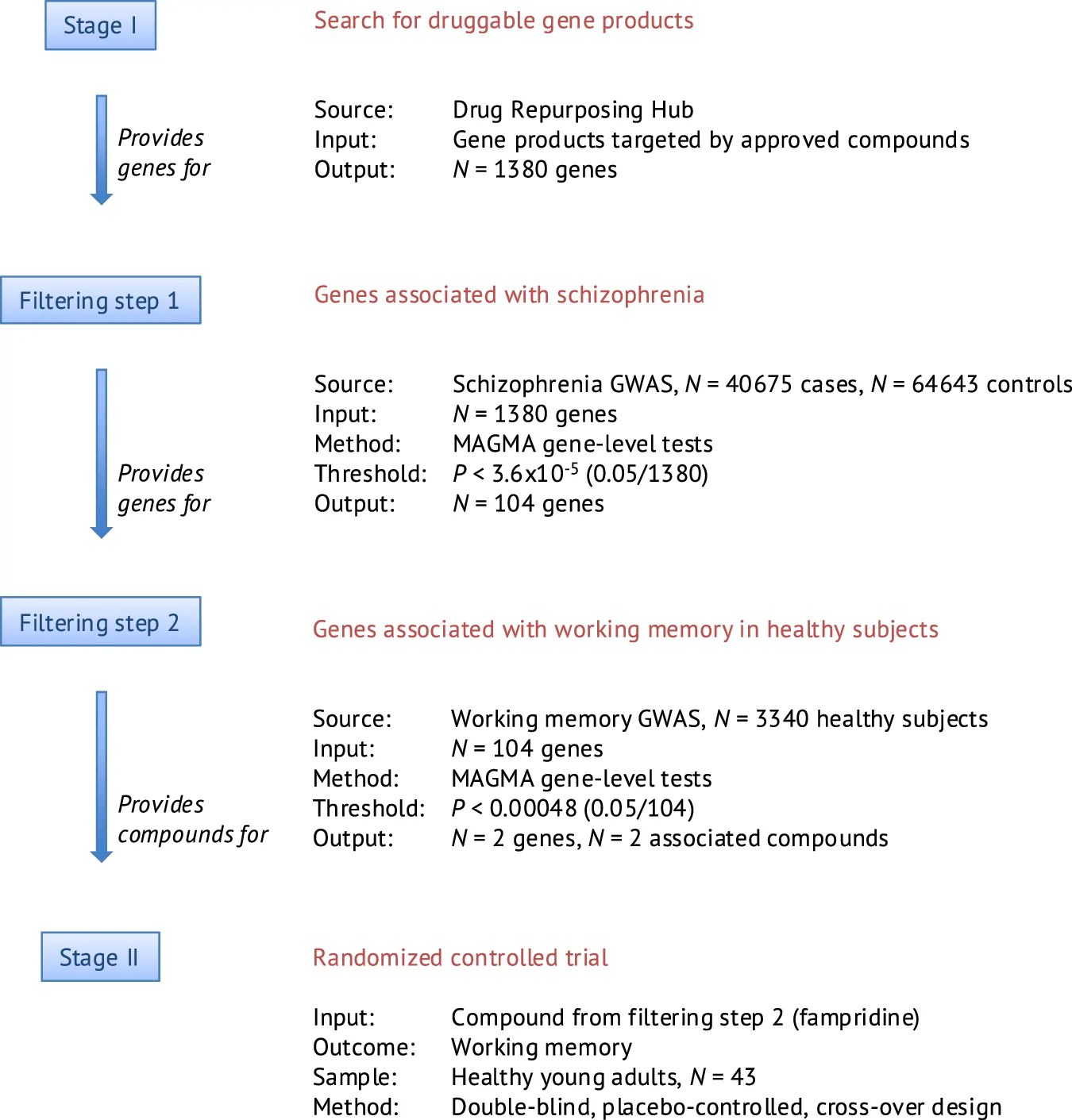
Drug selection. The aim of stage I was to identify approved compounds with a putative impact on WM performance, an established endophenotype of schizophrenia. In filtering steps 1 and 2, qualified genes (i.e., genes whose associated products are targets of approved compounds) concurrently implicated in the susceptibility to schizophrenia and in the execution of a validated WM task were identified. These steps provided candidate compounds for stage II, the randomized controlled trial.

Here, we used a genome-informed drug repurposing approach to nominate compounds predicted to affect WM performance and subsequently conducted a randomized controlled trial (RCT) with the most suitable compound (Fig. 1, see Drug Selection in the Results section). We initially identified all genes whose associated products are targets of approved compounds. The large schizophrenia GWAS [43], which provided genes robustly associated with the risk for the disorder, served as the first filtering step. This step enhances the probability of pinpointing statistically robust druggable targets. However, the GWAS identified genes associated with the overall risk of schizophrenia, some of which might be related to WM deficits, while others might be unrelated to cognitive symptoms. Therefore, we introduced a second filtering step, which encompassed the testing of schizophrenia-associated genes (that encode targets of approved compounds) for their association with WM performance in a sample of healthy young adults. Compounds targeting the products of genes which survived both filtering steps were considered for subsequent testing in an RCT. This procedure led to the nomination of fampridine (Fampyra®), a potassium channel blocker that is used to improve walking in adult patients with multiple sclerosis (MS) with walking disability [44], and to the conduct of an RCT that tested the potential of fampridine to modulate WM performance of healthy young adults.

## Participants and methods

### RCT design and participants

We conducted a double-blind, placebo-controlled, randomized, crossover trial comparing the effect of fampridine (Fampyra® 10 mg, given orally twice daily) with placebo. We recruited healthy participants from the Basel, Zurich, and Bern area of Switzerland through Internet advertisements. Forty-three participants entered the final analyses for the primary outcome (Supplementary Fig. S1), the respective number for the secondary outcomes was between 38 and 45 participants (see Table 1 for sample description). The experiment took place at the University of Basel. The study was conducted in accordance with the latest version of the Declaration of Helsinki. The local ethics committee approved the study (registration number: EKNZ, 2020-02828), and the study was pre-registered with ClinicalTrials.gov (NCT04652557) and kofam.ch (SNCTP000004227). All participants provided written informed consent and received a compensation of CHF 800 for their participation. Details on inclusion and exclusion criteria, randomization, blinding, RCT procedures, and RCT sample are provided in the supplement.

**Table 1:** Study sample description.

Number of participants	45
Age (years)	23.9 (3.2; 18–30)
Sex (female/male)	23/22
Body mass index (kg/m^2^)	23.6 (2.9; 19.2–29.8)
Body weight (kg)	71.5 (13.1; 51–107)
Resting heart rate (bpm)	68.6 (9.7; 48–92)
Systolic blood pressure (mmHg)	116.4 (10.6; 101–140)
Diastolic blood pressure (mmHg)	74.6 (6.4; 62–90)
Blood potassium (mmol/l)	4.0 (0.2; 3.6–4.4)
MADRS	1.4 (1.2; 0–5)

Values (assessed during the screening visit) are shown for all participants entering the analysis; values in parentheses represent standard deviation and range.

*MADRS* Montgomery–Åsberg Depression Rating Scale.

### RCT outcomes

The primary outcome measure was high-load working memory performance (3-back (d’) as assessed by a letter n-back task) after repeated intake (3.5 days) of the study medication. The 3-back task requires participants to respond to a letter repeat with two intervening letters (for example, s − m − b − s − g…). Performance was quantified with the d’ measure controlling for subjects’ response bias, i.e., the tendency to respond to target or non-target [45]. We used parallel versions (i.e., different sequences) for the test days.

We also predefined one non-behavioral and 7 behavioral secondary outcome measures.

Resting motor threshold (rMT) served as the non-behavioral secondary outcome. Behavioral secondary outcome measures were a.) High-load working memory performance (3-back (d’) as assessed by a letter n-back task, see above) after acute (i.e., after 4 h) intake of study medication on visits 2 and 4, b.) reaction time for correct 3-back responses after acute and repeated intake of study medication, c.) performance in a 0-back task (d’) after acute and repeated intake of study medication. Details on secondary outcome measures are provided in the supplement.

### RCT intervention

The active study medication comprised seven 10 mg tablets of fampridine formulated for oral administration, ingested twice daily at twelve-h intervals, preferably on an empty stomach. The tablets were to be swallowed whole without dividing, crushing, chewing, or dissolving the extended-release formulation of Fampyra®. A washout period of a minimum of eight days was implemented, exceeding 30 half-lives of the active moiety fampridine (t½ = 6 h), to mitigate any carryover effects between the experimental and control arms of the study. In January 2022, we amended the study protocol to extend the permitted washout period from 8–26 days to 8–82 days in anticipation of potential dropouts due to the SARS-CoV-2 Omicron wave. This modification allowed for greater flexibility in scheduling participants’ visits. The study utilized placebo tablets identical in appearance to the active medication, containing widely identical excipients, also formulated for oral administration. Details on adverse events are provided in the supplement.

### RCT statistical analyses

We were interested to detect a drug effect of at least medium effect size. A power analysis estimated a sample size of 44 (dependent two-tailed t-tests, Cohen’s d = 0.5, power 0.9 at α = 0.05, software: G*Power 3.1). Statistical analyses were performed in R (version 4.3.2).

Presuming Gaussian distribution properties of the primary and secondary outcome measures, the differences between the experimental conditions (placebo vs. active treatment) were pre-specified for analysis via linear mixed-effects models concomitant with Type II sum of squares factorial analysis of variance (ANOVA). Subjects were included as the random effect of the mixed model. Sex and age served as covariates. The significance threshold was set to *P* ≤ 0.05 for the primary outcome. For the behavioral secondary outcomes, we set the significant threshold to *P* ≤ 0.007 (Bonferroni correction for 7 independent tests). For the non-behavioral secondary outcome, the significance threshold was set to *P* ≤ 0.05. The study protocol explicitly allowed for additional, post-hoc analyses beyond the predefined ones in cases where effects known to potentially influence the primary outcome were identified. Further details on statistical analyses are provided in the supplement.

## Results

### Drug selection

We employed a drug repurposing strategy to identify compounds with a putative impact on WM performance, an established endophenotype of schizophrenia (Fig. 1). Leveraging the resources of the Drug Repurposing Hub (https://clue.io/repurposing-app) [46], we searched for all those genes whose associated products are targets of approved compounds. This search yielded 1380 genes (website accessed and data retrieved in June 2019). The large schizophrenia GWAS [43], which provided genes robustly associated with the risk for the disorder, served as an initial filtering step (i.e., we searched for genes pointing to approved compounds and being associated with schizophrenia). We performed gene-level tests in the schizophrenia GWAS data set using the MAGMA tool and the “multi” model, given its optimal statistical performance and sensitivity for a wide range of different genetic architectures [47]. We used a Bonferroni correction to define the gene level of significance, which was *P* < 3.6 × 10^−5^ (i.e., 0.05/1380). This analysis resulted in 104 genes whose products were targeted by a total of 452 approved compounds (Supplementary Dataset 1).

In the second filtering step, schizophrenia-associated genes encoding targets of approved compounds were tested for their association with WM performance (n-back task) in our incremental sample of deeply phenotyped healthy young subjects [14], that meanwhile comprised 3340 participants. We performed gene-level tests in this data set, and used again the MAGMA tool, the “multi” model, and a Bonferroni correction to define the gene level of significance, which was *P* < 0.00048 (i.e., 0.05/104 genes). This filtering step narrowed down the previous list of genes to two, *SLC2A2* (*P* = 2.3 × 10-5) targeted by the anti-cancer drug streptozotocin (US brand name Zanosar®, not available in Switzerland) and *KCNJ13* (*P* = 0.0001) targeted by fampridine (Swiss brand name Fampyra®), see also Supplementary Dataset 1 for detailed SNP association results. Fampridine (4-Aminopyridine) is a potassium channel-blocking agent with a long and varied history of applications, especially in neurology [48]. Fampridine, especially its slow-release formulation (Fampyra®), is generally a safe drug with well-studied pharmacokinetic properties [49]. It crosses the blood-brain barrier (BBB) and reaches maximum concentration in the brain approximately 3.5 h after single-dose administration [49]. Evidence suggests that fampridine improves walking speed in patients with multiple sclerosis (MS) [44], which led to Food and Drug Administration (FDA) and European Medicines Agency (EMA) approval for this indication. Some data also suggest that fampridine might have beneficial effects on cognitive functions in MS patients [50–52]. Given the genetic data provided herein and the available data on fampridine, we decided to conduct a placebo-controlled phase II cross-over study on the influence of fampridine on WM in healthy subjects (Supplementary Fig. S2).

### Effects of study medication on the primary outcome measure

**Fig. 2:**
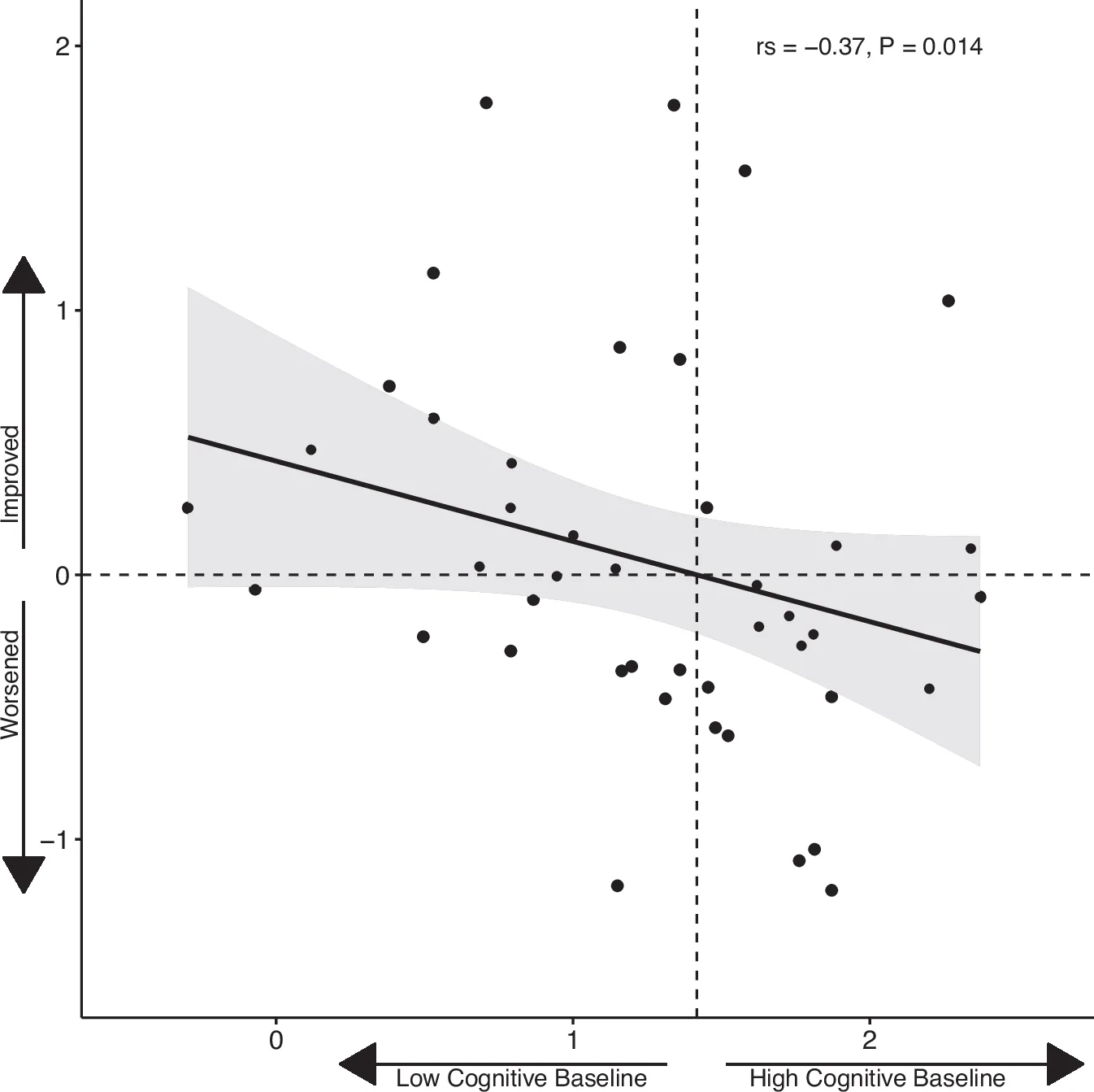
Correlation between individual measures of the primary outcome and individual baseline WM performance. Y-axis: individual difference (fampridine – placebo) in 3-back d’ after repeated administration; values above 0 represent improvement under fampridine, values below 0 represent decrease under fampridine. X-axis: individual baseline WM performance (3-back d’), the vertical dotted line represents the baseline performance level at which the regression line and the 0 level of the Y-axis intersect. The gray shaded area represents 95% confidence intervals of the linear regression line, drawn for visualization purposes. rs: Spearman’s rank correlation coefficient.

**Fig. 3:**
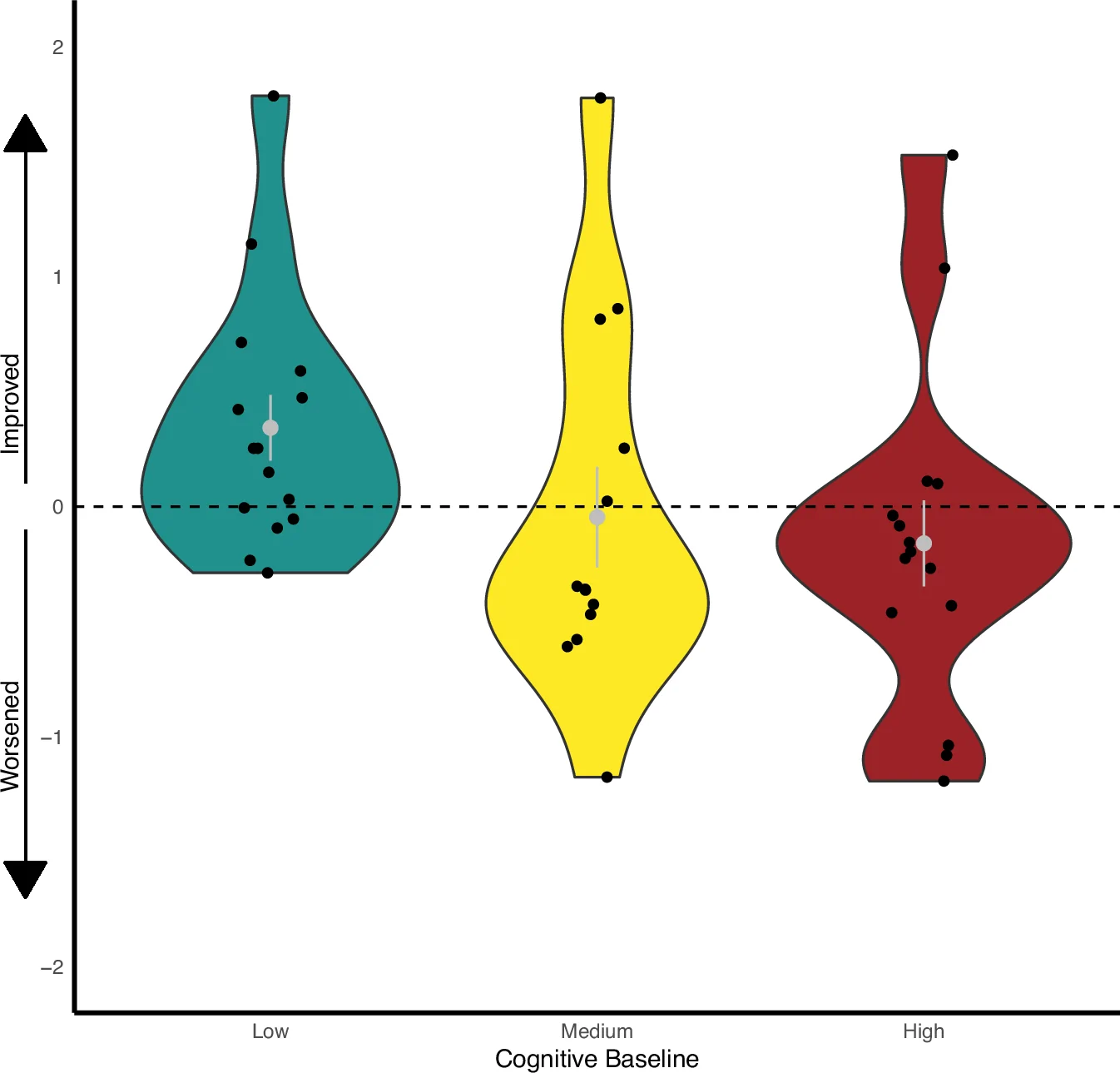
Post-hoc analysis of the drug effect on the primary outcome per cognitive baseline group, violin plots. Y-axis: individual difference (fampridine – placebo) in 3-back d’ after repeated administration; values above 0 represent improvement under fampridine, values below 0 represent decrease under fampridine. X-axis: baseline WM performance (3-back d’) stratified in three performance groups (i.e., low, medium, high terciles). Gray dots and vertical lines represent primary outcome measure means and standard errors of the means, respectively.

The primary outcome measure (i.e., high-load working memory performance (3-back d’) after repeated intake of the study medication) was not normally distributed (Shapiro–Wilk *P* = 0.046), hence non-parametric statistics (see RCT Statistical Analyses in Supplementary Material) were used for all analyses concerning the primary outcome. No significant main effect of the study medication was observed (Wilcoxon *P* = 0.87, r = 0.026, Table 2). This analysis was performed independently of baseline cognitive performance, which is known to significantly moderate the effect of pharmacological and non-pharmacological interventions on cognition [53–56]. We observed a significant negative correlation between baseline WM performance (i.e., high-load working memory performance after acute intake of placebo on visits 2 or 4, hence, assessed on a different day than the day of assessment of the primary outcome) and the primary outcome: lower baseline performance was associated with better WM performance after repeated intake of fampridine compared to placebo on visits 3 or 5 (r_s_ = −0.37, *P* = 0.014, *n* = 43; Fig. 2). A post-hoc analysis with the Jonckheere-Terpstra test confirmed this observation: a significant ordered effect of study medication after repeated administration across participants with low (i.e., low tercile), medium (i.e., middle tercile), and high (i.e., high tercile) baseline WM performance was detected (*z* = −2.24, r = −0.34, *P* = 0.025, *n* = 43). Post-hoc tests for each tercile separately revealed a significant main effect of the study medication for the low performance group, with fampridine resulting in better WM performance compared to placebo (r = 0.54, *P* = 0.035, *n* = 15; Fig. 3, Supplementary Fig. S3). No significant main effect of study medication was found in the medium (r = −0.17, *P* = 0.59, *n* = 13) or high baseline performance group (r = −0.40, *P* = 0.14, *n* = 15). Of note, the duration of the washout period did not affect the negative correlation between baseline WM performance and the primary outcome. Similar Spearman correlations were found for participants with a washout period <11 days and ≥11 days (median split; r_s_ = −0.41 and r_s_ = −0.38, respectively). The two washout period groups did not significantly differ in baseline performance (*P* = 0.55, Wilcoxon rank-sum test).

**Table 2:** Descriptive statistics of primary and secondary outcomes, effect sizes and significances.

Outcome	Administration	Condition	*N*	Mean	s.d.	*P* lme	*P* wilcox	r wilcox
3-back	repeated	Fampridine	43	1.55	0.67			
		Placebo	43	1.50	0.65			
		delta	43	0.05	0.71		0.872	0.026
3-back	acute	Fampridine	44	1.34	0.69			
		Placebo	44	1.28	0.66			
		delta	44	0.06	0.84	0.657		0.051
0-back	acute	Fampridine	44	3.70	0.22			
		Placebo	44	3.66	0.25			
		delta	44	0.04	0.31		0.508	0.142
0-back	repeated	Fampridine	43	3.64	0.34			
		Placebo	43	3.57	0.46			
		delta	43	0.07	0.53		0.323	0.099
rMT	repeated	Fampridine	38	61.61	12.01			
		Placebo	38	64.03	12.10			
		delta	38	−2.42	6.48	0.027		0.362
RT 3-back	acute	Fampridine	44	632.15	176.65			
		Placebo	44	629.96	203.33			
		delta	44	2.19	115.53	0.901		0.028
RT 3-back	repeated	Fampridine	43	583.79	172.91			
		Placebo	43	590.24	180.76			
		delta	43	−6.45	116.81		1.0	0.000
SDMT	acute	Fampridine	44	61.86	11.29			
		Placebo	44	63.09	8.18			
		delta	44	−1.23	10.23	0.431		0.114
SDMT	repeated	Fampridine	44	65.05	10.60			
		Placebo	44	68.34	10.84			
		delta	44	−3.30	11.42	0.062		0.280
BOMAT	acute	Fampridine	45	9.76	2.44			
		Placebo	45	9.76	2.71			
		delta	45	0.00	2.82		0.723	0.097
BOMAT	repeated	Fampridine	45	9.38	2.62			
		Placebo	45	9.91	2.69			
		delta	45	−0.53	3.29	0.283		0.161
ds forw	acute	Fampridine	43	8.19	1.85			
		Placebo	43	8.26	1.83			
		delta	43	−0.07	1.53		0.939	0.002
ds forw	repeated	Fampridine	45	8.38	1.99			
		Placebo	45	8.42	1.78			
		delta	45	−0.04	1.74		0.899	0.022
ds back	acute	Fampridine	45	8.60	1.95			
		Placebo	45	8.36	1.93			
		delta	45	0.24	1.81		0.445	0.151
ds back	repeated	Fampridine	43	8.72	1.88			
		Placebo	43	8.81	1.91			
		delta	43	−0.09	1.62	0.708		0.052

Outcome: 3-back and 0-back refer to 3-back (d’) and 0-back (d’), respectively, as assessed by a letter n-back task; rMT refers to resting motor threshold; RT 3-back refers to reaction time (milliseconds) for correct 3-back responses; SDMT refers to the Symbol Digit Modalities Test, its score represents the number of correct answers in 90 s; BOMAT refers to the Bochumer Matrizentest, its total score, with a possible range of 0 to 20, is calculated by summing up the number of correct responses; ds forw and ds back refer to the forward and backward digit span task, respectively, total scores were calculated as the sum of correct responses (0–12). Administration: acute refers to outcome measure 4 h after first intake of study medication; repeated refers to outcome measure after repeated intake (3.5 days) of the study medication. Condition: delta refers to the respective outcome value under fampridine less the outcome value under placebo. *P*lme: Significance calculated conditional on non-deviation from normality of the respective outcome. *P*wilcox: Significance calculated conditional on deviation from normality of the respective outcome.

### Effects of study medication on the non-behavioral secondary outcome measure

**Fig. 4:**
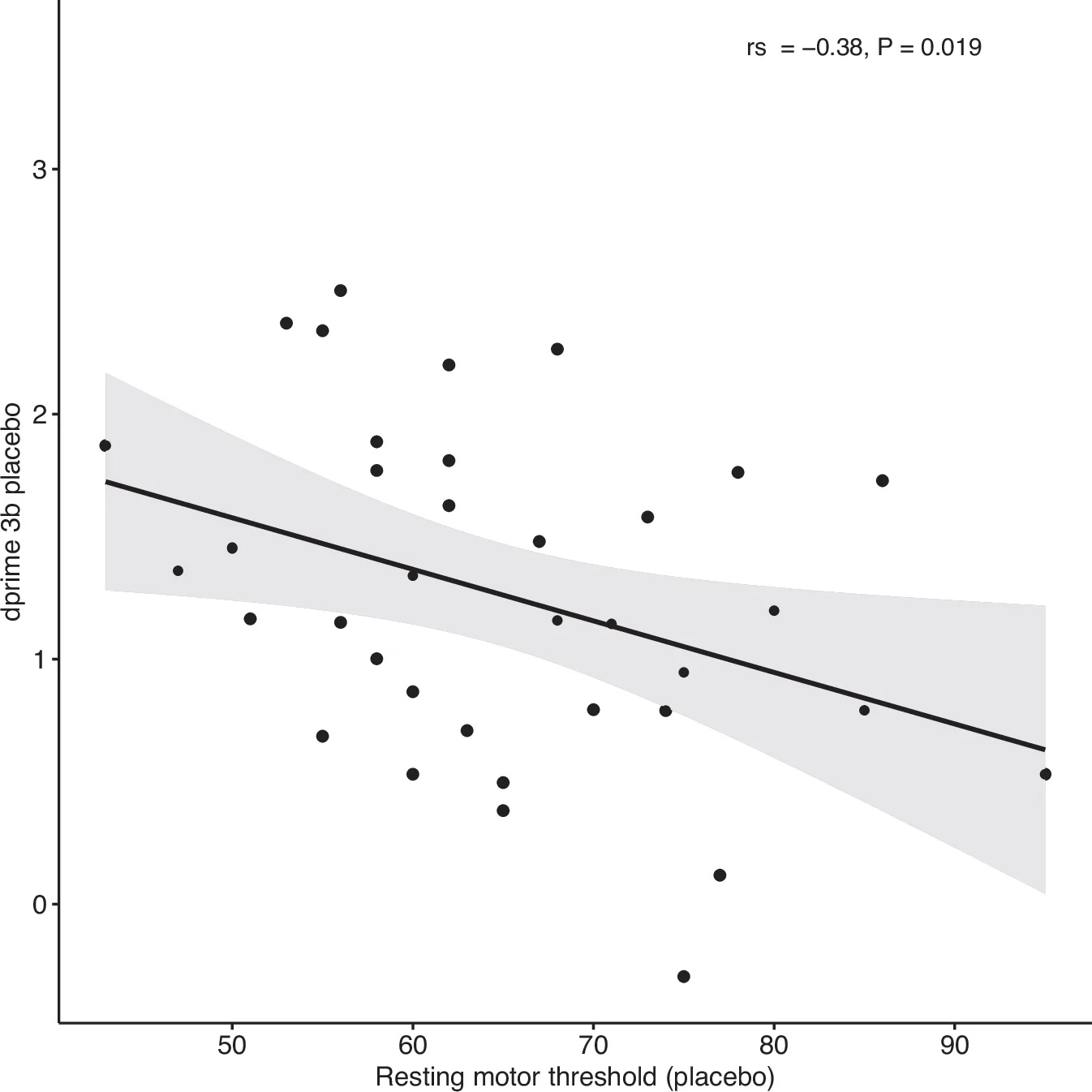
Correlation between resting motor threshold and WM performance. Y-axis: individual baseline WM performance (3-back d’ under placebo). X-axis: individual resting motor threshold under placebo. The gray shaded area represents 95% confidence intervals of the linear regression line, drawn for visualization purposes. rs: Spearman’s rank correlation coefficient.

There was a significant main effect of study medication on rMT after repeated intake (F(1,37) = 5.31, *P* = 0.027, R^2^β = 0.01, Table 2 for r wilcox), indicating decreased motor threshold under fampridine. A further analysis revealed that under placebo conditions, lower rMT was associated with higher baseline WM performance (r_s_ = −0.38, *P* = 0.019, *n* = 37; Fig. 4).

### Effects of study medication on the behavioral secondary outcome measures

There was no significant main effect of study medication on any of the behavioral secondary outcomes (nominal *P*s > 0.05, Table 2).

Fampridine was well tolerated, and all adverse events were mild to moderate in appearance and of short duration (see supplement).

## Discussion

The present study used genomic data to inform drug repurposing and to nominate approved drugs for testing on WM performance in the framework of an RCT. First, we searched for all those genes whose associated products are targets of approved compounds (i.e., qualified genes). Given our interest in physiological cognitive domains that are related to mental disease [14, 29, 57–59], our next objective was to delineate qualified genes concurrently implicated in the susceptibility to schizophrenia and in the execution of a validated WM task, an endophenotype of schizophrenia, among healthy individuals. This strategy pointed to potassium channels and to a related pharmacological compound, fampridine.

Potassium channels are salient pharmacological targets for heart and brain disorders [60] and offer additional opportunities for the development of new drugs to treat cancer, autoimmune diseases and metabolic disorders [61]. We and others have previously shown that potassium channels, including those targeted by fampridine, are genetic modifiers of WM performance, WM-related brain activity, risk for schizophrenia [14, 43] and brain connectivity [62]. In different sets of experiments in rodents, human, and non-human primates, pharmacological blockade of potassium channels has been shown to improve performance in cognitive assessments (for example refs. [50, 52, 63–65]).

The potassium channel-blocking agent fampridine (4-Aminopyridine), especially its slow-release formulation (Fampyra®), is generally a safe drug with well-studied pharmacokinetic properties [49] and approved for the symptomatic improvement of walking in MS [44]. Fampridine has also shown some beneficial effects on cognitive functions in MS patients [50–52]. Fampridine is recognized as a nonspecific antagonist of voltage-gated potassium channels and, with lower sensitivity, inward rectifying potassium channels. Consequently, multiple potassium channel genes are considered potential targets of fampridine [46] (see also Supplementary Dataset 1). One of the suggested modes of action (MOA) by which fampridine improves walking speed is its blockade of a spectrum of potassium channels that are exposed in demyelinated axons, leading to mitigation of potassium leakage and normalization of nerve conduction [66]. In addition to its long-term effects, fampridine also shows acute effects on nerve conduction [67]. Furthermore, an action of fampridine at central synapses and the potential augmentation of neurotransmitter release have been postulated [68, 69].

Continuous firing within specific neuronal circuits is central to WM functions [70]. Numerous studies performed mainly in rodents have shown that pharmacological and/or genetic inhibition of a variety of potassium channels, which are expressed in brain regions linked to WM, lead to increased neuronal excitability and improve performance in WM-related tasks (e.g., refs. [61, 71–75]). In the present RCT, we addressed the questions whether the potassium channel blocker fampridine affects WM and cortical excitability as reflected in resting motor threshold in healthy young subjects.

When considering the study population independently of individual baseline cognitive performance, no significant effect of fampridine was observed on WM performance. Importantly, baseline cognitive performance has been shown to have a significant impact on pharmacological and non-pharmacological interventions on cognition. A recent placebo-controlled study on the effect of methylphenidate on cognition in children with ADHD addressed specifically this question. The degree of WM enhancement observed following medication was most pronounced in children exhibiting poorer WM performance at baseline. Conversely, children demonstrating higher WM performance prior to intervention displayed a diminished or null response in WM increase when treated with different doses of methylphenidate [53]. That study is in line with earlier observations in healthy adults showing that dextroamphetamine enhances performance exclusively in subjects with relatively low baseline WM capacity, while it impairs performance in those with high baseline WM capacity [55]. It is also in line with studies suggesting that, in healthy adults, baseline WM capacity correlates negatively with the magnitude of WM improvement following methylphenidate administration [56], and that amphetamines enhance inhibitory control predominantly in subjects characterized by low baseline inhibitory function [76]. These observations extend also to non-pharmacological enhancement of cognitive function. In a recent study of cognitively healthy older adults that tested the effect of repetitive transcranial neuromodulation on cognitive performance, individuals with lower baseline cognitive function exhibited larger and more enduring memory improvements [54]. We observed a significant negative correlation between baseline WM performance and the drug effect on the primary outcome measure: lower baseline performance was associated with better WM performance after repeated intake of fampridine compared to placebo. After post-hoc stratification of the study population in groups exhibiting different baseline WM performance, a significant WM-enhancing effect of fampridine was observed only in the lowest baseline performance group. The existing evidence and the data of the present study indicate that in trials where pharmacological interventions are expected to yield small to moderate effects (as is the case in most psychopharmacological treatments), the consideration of moderating variables at the individual level becomes crucial for elucidating differential responses to treatment [53]. The data might also imply a need for dose stratification based on baseline cognitive abilities, assuming that individuals with lower cognitive performance at baseline might benefit from different dosing regimens compared to those with higher initial cognitive function to optimize cognitive enhancement. Additional studies are needed to test whether these considerations extend to fampridine’s influence on WM performance.

In addition to its effect on WM performance after repeated administration, fampridine significantly reduced participants’ resting motor threshold (rMT). rMT, mediated by ion channel conductivity, provides an objective measure of cortical excitability [77] that is per se linked to cognitive functions [70]. Importantly, lower rMT has been linked to better WM performance in healthy adults [78], a finding that was replicated in the present study.

The choice of fampridine and of the primary outcome measure were based on robust observations. Nevertheless, our study is confronted with challenges that are inherent to any clinical trial. Importantly, the selected dose might have been too high or too low. On the other hand, it corresponds to the daily dose approved by the FDA and EMA in MS patients, and we were restricted to this regimen for this repurposing RCT. Further, the repeated administration was restricted to 3.5 days and we cannot make any conclusions with regard to longer treatment periods.

While the use of a healthy, young cohort (Table 1) was appropriate for the proof-of-concept nature of this study, it inherently limits the generalizability of our findings to broader, more heterogeneous populations. Given the potential variability in response across different demographic groups, particularly in older adults or individuals with comorbidities, further studies are warranted to explore the efficacy and safety of the intervention in more diverse samples. Nevertheless, our approach is in line with NIMHs Fast-Fail Trials (FAST) initiative, which aims at identifying targets in the brain at which an intervention, like a pharmaceutical, could be directed to correct a specific dysfunction contributing to mental illness. The difference is that we tested the drug in healthy participants first, before embarking on larger and more detailed studies addressing patients with WM-related mental disorders, such as schizophrenia. With respect to fampridine’s MOA, the restoration of conduction in demyelinated axons is just one of the suggested mechanisms through which the drug alleviates MS symptoms. Importantly, numerous experiments support additional MOAs not involving the demyelinated portions of axons. For example, fampridine rapidly potentiates neuro-neuronal synaptic transmission, an effect that involves potassium channel blockade-dependent increase in neurotransmitter release and an increase in the number of synaptic terminals activated by an action potential (for example refs. [49, 79, 80]). Given the drug’s effect on myelination-independent synaptic transmission and the fact that working memory depends on the continuous firing within neuronal circuits [70], there is a reasonable possibility that fampridine improves WM through these mechanisms.

The genome-based drug repurposing strategy delineated in this report exemplifies one among multiple possible methodologies employable for harnessing genomic data in drug discovery and development. In this study, the selection process for compounds to enter the RCT prioritized operational feasibility, restricting consideration to compounds registered and commercially available in Switzerland. Moreover, the selection criteria might have been too rigorous, mandating Bonferroni adjustment during the filtering phases (Fig. 1). This rigorousness potentially excluded several promising agents (Supplementary Dataset 1) that may be suitable for future RCTs. A comprehensive investigation into the translational and repurposing capacity of genomic data would necessitate the application of alternative filtering criteria.

In summary, we report that the influence of fampridine on WM performance in healthy individuals depends on their baseline WM performance. In addition, fampridine reduced resting motor threshold, an objective measurement and correlate of cortical excitability linked to cognitive performance.

The observation that individual baseline cognitive function moderated the cognitive response to fampridine and that the drug had a pro-cognitive effect in individuals with low WM performance point to a potential value of the drug in treating WM deficits in psychiatric disorders. Importantly, cognitive deficits, in particular WM deficits, are a common culprit of schizophrenia [3–6] and bipolar disorder [7], and represent some of the most pronounced and disabling clinical manifestations [81]. Despite ongoing pre-clinical and clinical developments, current pharmacological interventions are still inadequate for ameliorating the cognitive deficits associated with these disorders [82]. In this respect, targeting genetic markers that exhibit pleiotropic effects across multiple psychiatric disorders may be a valuable strategy for identifying genes with transdiagnostic therapeutic potential in the context of genome-guided drug discovery. However, this approach is limited by the differing sample sizes of individual GWAS, which affect the power to detect genetic markers for each disorder. As GWAS sample sizes increase and become comparable across various disorders, applying statistical methods that leverage potential pleiotropy between carefully selected psychiatric disorders and endophenotypic traits may hold promise for identifying novel genomic loci, potentially revealing drug repurposing candidates.

Recent data indicate that treatment-related cognitive improvements might be confined to individuals exhibiting significant baseline cognitive deficits [82]. The results of the present study support these considerations and might initiate pro-cognitive treatments with known and potentially also new and specific potassium channel blockers in psychiatric patients. In our view, an RCT assessing a possible WM-enhancing effect of fampridine in schizophrenia is warranted. It will be also worthwhile to consider the potential for a pharmacogenetic approach to investigate the interaction between fampridine and potassium channel-specific gene variations. Such a strategy could be valuable in identifying patient sub-groups with an increased likelihood of drug efficacy or a reduced risk of adverse events. However, some considerations would have to be addressed before initiating such pharmacogenetic studies. First, it would be advantageous to prioritize genetic variants with well-defined functional consequences on gene expression or protein function, rather than relying on marker SNPs with no known mechanistic impact. Such variations are still to be identified. Second, while these variants may help to delineate sub-groups with distinct therapeutic responses, such stratification will necessitate larger clinical trial cohorts to ensure sufficient power for detecting subgroup-specific effects.

The choice of fampridine in this study was a result of a repurposing strategy guided by genomic information. We anticipate that the rapidly increasing genetic knowledge of psychiatric disorders together with the use of biology-informed phenotypes and appropriate data-mining methodology will be a starting point for the identification of novel drug targets and treatments in psychiatry.

## Supplementary information

Supplemental material

Supplementary Dataset 1

## Data Availability

The data that support the findings of this controlled trial are not openly available due to reasons of participant privacy and are available from the corresponding author upon reasonable request. Data are located in controlled access data storage at the University of Basel.
