# Supplementary material for: The effect of fampridine on working memory: a randomized controlled trial based on a genome-guided repurposing approach
Source: Mol Psychiatry. 2024 Nov 8;30(5):2085–94. doi: 10.1038/s41380-024-02820-1 (PMC12014476; doi:10.1038/s41380-024-02820-1)
Supplement: Supplementary file 1 — Supplemental material [file 41380_2024_2820_MOESM1_ESM.docx]

Supplementary Material

Participants and Methods

*RCT design and participants*

To meet the inclusion criteria, participants had to be between 18 and 30 years old, have a body mass index (BMI) between 19 and 30 kg/m2, be native or fluent German-speaking, be normotensive (blood pressure between 90/60 mmHg and 140/90 mmHg), and have been vaccinated at least twice against COVID-19. Exclusion criteria were one or more of the following conditions as assessed with a self-report health questionnaire or during clinical examination: known hypersensitivity or allergy to 4-aminopyridine, use of potassium channel blockers within the last 3 months, concomitant treatment with organic cation transporter 2 (OCT2) inhibitors and substrates (e.g., cimetidine, propranolol), acute or chronic psychiatric disorder (e.g. major depression, psychoses, somatoform disorder, suicidal tendency), acute cerebrovascular condition, history of seizures, risk of lowered seizure threshold (e.g., due to sleep deprivation, withdrawal of alcohol after alcohol abuse), renal impairment, history of malignant cancers, walking problems (e.g. due to dizziness), other clinically significant concomitant disease states (e.g., hepatic dysfunction, cardiovascular disease, diabetes, asthma), bradycardia < 50/min, clinically significant laboratory or ECG abnormality that could be a safety issue in the study, known or suspected non-compliance, drug or alcohol abuse, inability to follow the procedures of the study, participation in another study with an investigational drug within the 30 days preceding and during the present study, prior participation (less than two years ago) in a study investigating working memory using the n-back task, being related to or in any form dependent on the investigator, smoking (>3 cigarettes per day), intake of psychoactive drugs (e.g. benzodiazepines, antidepressants, neuroleptics), pregnancy or breast feeding. Exclusion criteria related to the measurement of resting motor threshold (rMT) by means of transcranial magnetic stimulation (TMS) were: previous experience of a syncope during rMT measurement, presence of metallic material in the body (e.g., splinters, fragments, clips, etc.), implanted neurostimulator of any form, cardiac pacemaker or intracardiac lines, medication infusion device, piercings, pivot teeth (retainers were not an exclusion criterion), tattoos (head area) less than 3 months old or older than 20 years, past neurosurgery, hearing problems or tinnitus, not able to sit still due to tremor, tics, itching, history of repeated syncope, history of head trauma diagnosed as concussion or associated with loss of consciousness, diagnosis of epilepsy, or a convulsion or a seizure in the past of the participant or his first-degree relatives, TMS in the past showing problems, MRI exam in the past showing problems, spinal cord surgery, and spinal or ventricular derivations.

*Randomization and blinding*

Upon pre-examination, participants were conferred a unique randomization identifier. Subsequently, eligible subjects were allocated to one of two stratification groups—placebo/active treatment or active treatment/placebo—employing the Maximum Tolerated Imbalance (MTI) strategy. This allocation schema was facilitated through an automated process within the secuTrial® software platform (interActive Systems GmbH, Berlin, Germany), which inherently incorporated sex as a stratifying variable. Following the full assignment of 44 medication kits, any participant attritions were substituted, an iterative process perpetuated until the achievement of a complete dataset from 44 distinct participants.

The Investigational Medicinal Product (IMP) and placebo were provided as visually indistinguishable tablets to maintain double-blind conditions. The pharmacy prepared a medication kit for each participant, comprising two opaque plastic containers. One container held seven blister-packed fampridine tablets with a desiccant, while the other contained unblistered placebo tablets with a desiccant. To mitigate fampridine's exposure to humidity, Fampyra® was retained in its blister packaging until administration. Once deblistered, Fampyra® remained stable for seven days in a sealed container with a desiccant, per the professional prescribing information in Germany for Fampyra® SR. Each medication kit was assigned a unique randomization number. An individual not involved in the study's execution opened the kit and the appropriate container in a separate room. If the container held active drug, the individual deblistered the tablets first and then administered the first dose to the participant in an adjacent room. If the container held unblistered placebo, the first tablet was provided to the participant in an adjacent room. Participants were then given the resealed container to continue medication intake at home, consuming one tablet every 12 hours for five doses. The seventh and final dose was to be taken at the study center, requiring participants to return the container with the last tablet on the second day of each intervention period.

Investigators conducting the test sessions underwent training and adhered to detailed operational guidelines, thereby ensuring a high degree of standardization across test days. Blinding was implemented for study personnel engaged in testing, data collection, and analysis. The individual responsible for preparing the IMP during test days was segregated from other outcome-related study duties.

Individual participant testing occurred at the Division of Cognitive Neuroscience, University of Basel, and at a consistent time of day to control for diurnal variations in cognitive performance.

*RCT procedures*

Recruitment of study participants was conducted in the German-speaking region of Switzerland via the websites mcn.unibas.ch and markt.unibas.ch. Interested individuals who contacted our team were provided with "Participant Information and Informed Consent" documents alongside administrative details. Those maintaining interest were engaged via telephone by a study team member who provided detailed study information and addressed any inquiries. A subsequent pre-screening to verify the primary inclusion and exclusion criteria was executed. Candidates meeting all inclusion criteria, none of the exclusion criteria, and expressing a willingness to participate were scheduled for a screening visit (Supplementary Fig. S2).

Prospective participants were instructed to refrain from visiting the study center if experiencing symptoms indicative of a common cold, influenza, or COVID-19, and to reschedule accordingly. Documentation from the telephone screenings was incorporated into the Source Data within each participant’s dossier. Records pertaining to individuals deemed ineligible for study participation were destroyed. Comprehensive documentation was maintained throughout the trial.

During the screening visit, the investigator elucidated the study's objectives, procedures, investigational drug, and associated risks to the participant. Written informed consent was obtained from each participant, who were given unlimited time for contemplation and signature. Eligibility for study enrollment was determined through assessments conducted by the investigator, based on inclusion and exclusion criteria. The screening visit occurred within a fortnight preceding the initial test visit and included personal and familial history evaluation, psychosocial assessment, medication history review, and physical examination.

The presence of psychiatric disorders was assessed using the MADRS-S, a self-rating scale version of the MADRS (Montgomery-Åsberg Depression Rating Scale) [1], and a self-assessment questionnaire capturing mental health status, substance use, sleep patterns, and sociodemographic data. MADRS-S encompasses nine items that evaluate mood, unease, sleep, appetite, concentration, initiative, emotional involvement, pessimism, and life enthusiasm. Items were scored from 0 to 3, with a cumulative score ranging from 0 to 27, where higher scores signified greater impairment. Participants scoring 8 or above, or exhibiting suicidal tendencies (Item 9 > 1), were excluded and referred for psychological or psychiatric consultation with provided contacts.

The investigator determined any contraindications to study medication administration. Medical examinations included vital sign measurement after a 5-minute seated rest, body-mass-index (BMI) calculation, a handedness questionnaire (short Edinburgh Handedness Inventory), physical examination, twelve-lead ECG (Wilson, Einthoven, Goldberger) conducted with a Schiller AT10 device, interpreted by a specialist at the Phase I Research Unit (PRU), University Hospital Basel, and venous blood samples for comprehensive laboratory analysis. Abnormal, clinically significant results led to participant exclusion.

Resting Motor Threshold (rMT) determination was performed as described below.

Female participants of childbearing potential were instructed to employ effective contraception beginning ten days prior to and during intervention periods. Pregnancy tests were mandated before study drug administration on visits 2 and 4 to mitigate IMP exposure risks. Screening failures, defined as non-fulfillment of all inclusion criteria or meeting any exclusion criteria, were logged. The screening process was reiterated if the washout period exceeded 26 days.

On visits 2 and 4, female participants underwent pregnancy testing using a qualitative urine hCG test. A brief examination recorded vital signs, concomitant medication, sleep duration, substance use, and adverse events. Medication was administered at the same time of day to all subjects. To minimize diurnal cognitive performance variances, testing was uniformly scheduled. A 4-hour latency allowed for peak cerebrospinal fluid drug concentrations, followed by a cognitive test battery. Participants’ wellness was inquired at day's end, with medical oversight for any health concerns. Study tasks, excluding laboratory and ECG evaluations conducted at the PRU, including medication administration and outcome assessments, were carried out at the Research Platform Molecular and Cognitive Neurosciences, University of Basel.

At the end of visits 2 and 4 participants were dispensed a container with six remaining investigational product tablets for bi-daily administration during subsequent days, with detailed intake and adverse event documentation within secuTrial®.

*RCT sample*

Between November 8, 2021, and January 3, 2023, 71 individuals were screened for trial participation (Supplementary Fig. S1). Twenty-one subjects were excluded after screening. Consequently, 50 individuals were included and underwent randomization for treatment order. Twenty-six subjects were allocated to receive fampridine first, 24 to receive placebo first. Four subjects dropped out and did not participate in the second treatment phase (the reasons were: in 3 subjects, the wash-out phase was too long due to the study interruption caused by the SARS-CoV-2 Omicron wave, one participant was involved in a car accident as a passenger the night before the end of the first treatment phase). The actual duration of the washout period ranged from 9 to 45 days (mean (s.d.), 15.8 (7.8) days). Two of the 45 participants exceeded the originally planned washout period (45 and 31 days, respectively; see Table S1). Forty-six subjects underwent the procedures of the second treatment phase, 23 received fampridine and 23 received placebo. Complete data of 46 subjects were available. Before unblinding, one participant was excluded due to non-compliance for all outcome assessments. Therefore, data of 45 subjects entered the analyses. Participants’ age range was 18-30 years (mean (s.d.), 23.9 (3.2) years), 23 participants were female. Body-mass-index (BMI) ranged from 19.2 kg/m2 to 29.8 kg/m2 (mean (s.d.), 23.6 (2.9) kg/m2), body weight ranged from 51 kg to 107 kg (mean (s.d.), 71.5 (13.1) kg). For the primary outcome, two more subjects were excluded before unblinding (one due to non-compliance during the n-back task and one because the task had not run through completely, Supplementary Fig. S1).

*RCT Secondary Outcomes*

We predefined one non-behavioral and 7 behavioral secondary outcome measures.

Resting motor threshold (rMT) served as the non-behavioral secondary outcome. rMT, mediated by ion channel conductivity, provides an objective measure of cortical excitability[2] that is *per se* linked to cognitive functions[3]. rMT was assessed during screening and following repeated administration using a biphasic Magstim Rapid2 stimulator (The MAGSTIM® Company Ltd, Whitland, UK) equipped with a 70 mm figure-of-eight coil.

rMT was determined by measuring the motor evoked potential (MEP) in the abductor digiti minimi muscle [4]. Specifically, rMT was defined as the minimum intensity needed to evoke an MEP with a peak-to-peak amplitude exceeding 50 µV in at least 5 out of 10 consecutive trials in the relaxed dominant hand. Positioning of the coil was guided by the Brainsight frameless stereotaxis neuronavigation system. During the screening visit, the hotspot was initially located by placing the coil tangentially to the scalp over the hand area representation of the primary motor cortex (M1), with the coil handle oriented backwards at a 45° angle relative to the parasagittal line. The coil position was then systematically adjusted in 1 cm increments in anterior, posterior, lateral, and medial directions. Stimulation intensity started with 30% of the maximum stimulator output (MSO). If no MEP > 50 µV or visual muscle twitch in any hand muscles were observed, MSO was increased by 10%. If MEP > 50 µV was observed, the position of the coil was saved in Brainsight and again the coil was systematically moved until the best position was found. In case of doubt, the intensity was increased by another 5% to determine the best location. This position was then saved as individual hotspot and reused in visit 3 and 5.

To minimize participants' focus on hand muscle contractions, they were instructed to count backwards loudly from 200 in steps of 1 and to look at a picture on the wall during the procedure. TMS pulses were then applied at the hotspot position with intervals of more than 5 seconds and at irregular intervals. If at least 5 MEPs with a peak-to-peak amplitude greater than 50 µV were observed, the intensity was decreased by 5%. Depending on the response, the intensity was further adjusted in steps of two and then one percent until the lowest intensity meeting the criteria was found. Finally, the intensity was reduced by an additional 1% to confirm that it did not meet the requirements.

During visits 3 and 5, rMT determination started with the previously saved hotspot position of the screening visit at an intensity of 30%. Participants retained the right to discontinue the measurement if discomfort was experienced.

Behavioral secondary outcome measures were a.) High-load working memory performance (3-back (d’) as assessed by a letter n-back task, see above) after acute (i.e., after 4h) intake of study medication on visits 2 and 4, b.) reaction time for correct 3-back responses after acute and repeated intake of study medication, c.) performance in a 0-back task (d’) after acute and repeated intake of study medication. We used parallel versions (i.e., different sequences) for the four test days. Unlike the 3-back task, where participants must indicate when the current stimulus matches the one from 3 steps earlier in the sequence, the 0-back condition requires participants to respond to the letter “x”. The 0-back task served as a measurement of baseline attentional processes, as it minimally engages the working memory systems involved in updating, maintenance, and manipulation of information, d.) the Symbol Digit Modalities Test (SDMT)[5], a processing speed test, after acute and repeated intake of study medication. The test consists of the presentation of a series of 9 symbols, each of them is paired with a single digit, labelled 1–9, in a key at the top of a sheet. The remainder of the page has a pseudorandomized sequence of the symbols, and the participant must respond with the digit associated with each of these as quickly as possible. The score represents the number of correct answers in 90 seconds. The administration of SDMT was preceded by a learning sequence at both timepoints. We used parallel versions for all test days, e.) the Bochumer Matrizentest (BOMAT – advanced)[6], matrix reasoning, after acute and repeated intake of study medication. We administered the BOMAT to assess fluid intelligence (Gf), encompassing 40 items. Parallel versions were used for the four test days. To create four versions, we divided the original BOMAT versions A and B each into two versions (every second item) with 20 items each. We used a time-limited version (15 instead of 40 minutes) to avoid a ceiling effect. The total score, with a possible range of 0 to 20, was calculated by tallying the number of correct responses, f.) the digit span task, forward and g.) backward, a subtest of the “Wechsler Intelligenztest für Erwachsene“ (WIE)[7] after acute and repeated intake of study medication. Total scores for digit span forward and backward were calculated as the sum of correct responses (0-12). Parallel versions were used for the four test days.

*RCT statistical analyses*

Presuming Gaussian distribution properties of the primary and secondary outcome measures, the differences between the experimental conditions (placebo vs. active treatment) were pre-specified for analysis via linear mixed-effects models concomitant with Type II sum of squares factorial analysis of variance (ANOVA). Subjects were included as the random effect of the mixed model. Sex and age served as covariates. To control for possible confounders or moderators, we considered tiredness, motivation, headache, gastrointestinal discomfort, dizziness, mood as assessed by MADRS-S, potassium blood concentration at screening, body weight, duration of washout period and vital signs as covariates (each covariate entering the statistical analysis separately). In case of a significant (i.e., after Bonferroni correction for multiple comparisons) interaction between the independent variable covariates, post-hoc analysis was applied to describe the interaction effect. For secondary behavioral outcomes, a Bonferroni correction (i.e., corrected for the number of behavioral secondary outcomes) applied separately for the main effect (i.e., active medication vs. placebo) and the interaction effect (i.e., main effect x acute vs. repeated administration). In the case of a significant (i.e., Bonferroni-corrected) interaction effect, post-hoc analysis was applied to describe the interaction effect. The Bonferroni correction was pre-specified for the secondary behavioral outcomes only.

The Shapiro-Wilk test, which is particularly suitable for sample sizes <50, was used to test for normality[8]. In case of violation of the normality assumption (i.e., Shapiro-Wilk *P*<0.05), the non-parametric Wilcoxon test for paired samples was used to analyze differences between the experimental conditions (placebo vs. active treatment), whereby the dependent variables were residualized for sex and age. Wherever applicable, non-parametric Spearman’s ρ was calculated to describe correlations between variables that deviated from normality. The non-parametric Jonckheere-Terpstra test was used to test for trends across more than two independent samples. The employment of non-parametric testing was not pre-specified in the protocol but was deemed a requisite statistical adjustment in instances of significant deviations from normality.

A data monitoring committee oversaw the study. A clinical trial monitor oversaw data collection and entry according to a written monitoring plan, which was approved by the Independent Ethics Committee before the start of the trial.

*Adverse events (AEs)*

Fifty subjects received at least one dose of medication (fampridine or placebo) and were eligible for safety analysis. In this study, following very common AEs were excluded from recording but were measured as possible confounding factors: headache, gastrointestinal disturbances such as dyspepsia, abdominal pain, constipation and nausea, and dizziness.

All other AEs were fully recorded by describing the AE, time of onset, duration and resolution, assessment of intensity, relationship to study drug and measures taken.

No serious AE (SAE) related to study medication occurred. No subject withdrew from the study. One subject had to be excluded due to involvement in a car accident as a passenger in the night before test visit 2. Four subjects experienced a total of 5 adverse events during the study, 2 AEs occurred during fampridine application (shoulder contusion as a passenger in a car accident, neck pain), 3 during placebo application (dizziness, pain when chewing, toothache). These adverse events were unlikely related to drug. All AEs were mild to moderate in appearance and of short duration.

**Table S1. Information on the washout period of study participants**

| **Subject #** | **Sex** | **Started with** | **Duration of washout period (days)** |
| --- | --- | --- | --- |
| 1 | male | Placebo | 9 |
| 2 | male | Placebo | 9 |
| 3 | female | Placebo | 10 |
| 4 | female | Placebo | 10 |
| 5 | male | Placebo | 10 |
| 6 | female | Placebo | 10 |
| 7 | female | Placebo | 10 |
| 8 | female | Placebo | 10 |
| 9 | female | Placebo | 10 |
| 10 | female | Placebo | 10 |
| 11 | female | Placebo | 10 |
| 12 | male | Placebo | 11 |
| 13 | male | Placebo | 15 |
| 14 | male | Placebo | 17 |
| 15 | male | Placebo | 24 |
| 16 | male | Placebo | 24 |
| 17 | female | Placebo | 24 |
| 18 | male | Placebo | 24 |
| 19 | female | Placebo | 24 |
| 20 | male | Placebo | 24 |
| 21 | male | Placebo | 31 |
| 22 | female | Placebo | 45 |
| 23 | male | Verum | 9 |
| 24 | male | Verum | 10 |
| 25 | female | Verum | 10 |
| 26 | female | Verum | 10 |
| 27 | male | Verum | 10 |
| 28 | male | Verum | 10 |
| 29 | male | Verum | 10 |
| 30 | female | Verum | 10 |
| 31 | female | Verum | 10 |
| 32 | female | Verum | 10 |
| 33 | female | Verum | 10 |
| 34 | female | Verum | 11 |
| 35 | male | Verum | 11 |

**Table S1 (continued). Information on the washout period of study participants**

| **Subject #** | **Sex** | **Started with** | **Duration of washout period (days)** |
| --- | --- | --- | --- |
| 36 | male | Verum | 16 |
| 37 | male | Verum | 17 |
| 38 | male | Verum | 17 |
| 39 | male | Verum | 18 |
| 40 | female | Verum | 23 |
| 41 | female | Verum | 23 |
| 42 | female | Verum | 24 |
| 43 | female | Verum | 24 |
| 44 | female | Verum | 24 |
| 45 | male | Verum | 25 |

**Fig. S1 Consolidated Standards of Reporting Trials (CONSORT) flow diagram.**

**Fig. S2 Study design overview.** Illustration of the double-blind, placebo-controlled, randomized, crossover design of the study. Following a screening visit (Visit 1), included participants were randomized to receive either fampridine or placebo first. At Visits 2 and 4, cognitive testing was conducted 4 hours post-administration of the initial dose of either fampridine or placebo. Visits 3 and 5 involved cognitive testing at the same time of day as at Visits 2 and 4, and measurement of the resting motor threshold, occurring 4 hours after the final dose, following a 3.5-day period of repeated administration. This protocol ensured consistent timing for cognitive assessments and maintained elevated plasma levels of the medication on the final day of testing.

*
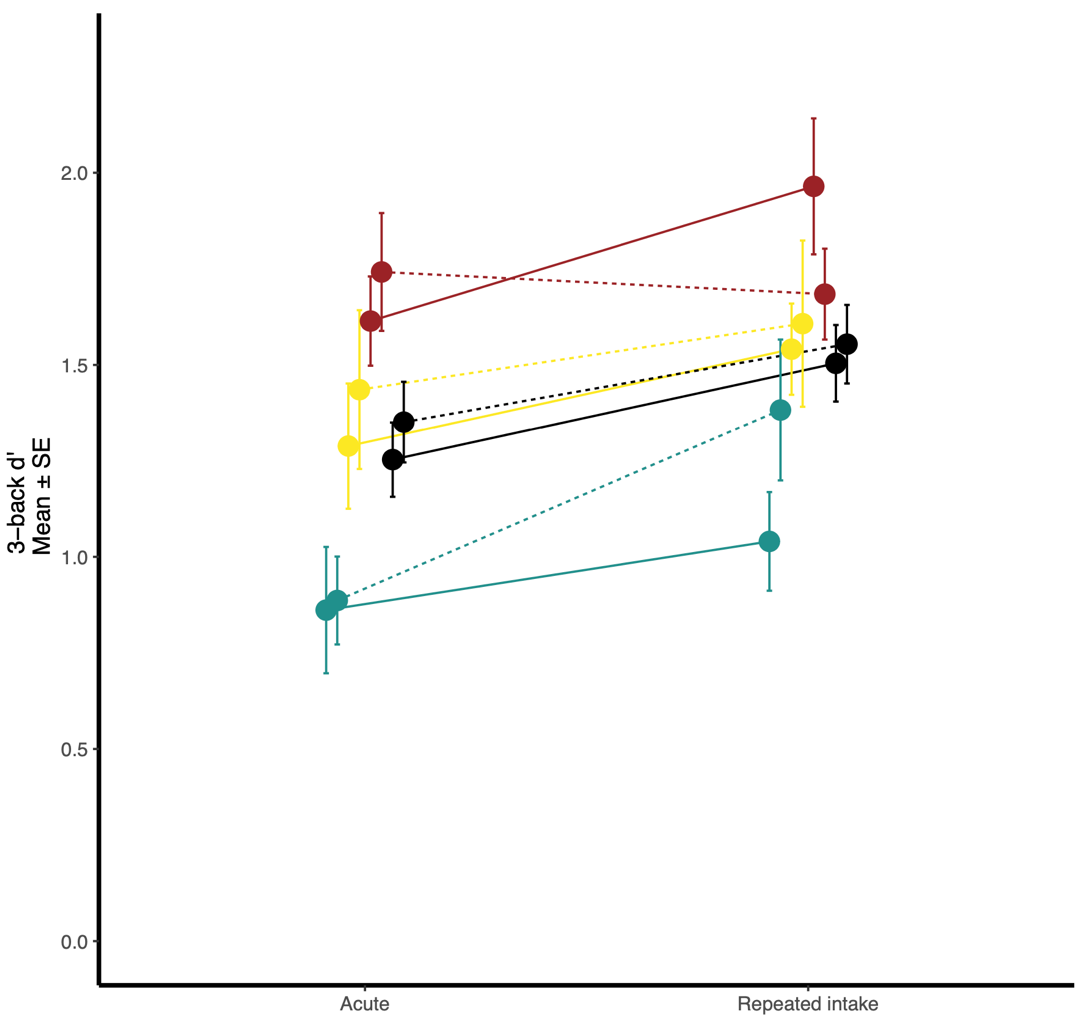
*

**Fig. S3 Time course of cognitive performance (3-back d’, primary outcome) after acute and repeated intake (3.5 days) of the study medication.** Black color represents the entire, unstratified sample. Green, yellow, and red represent the low, medium, and high terciles of baseline performance, respectively, as assessed on a day different from the testing day. Continuous lines represent the placebo phase, dashed lines represent the drug (Fampridine) phase.

References

1 Schmidtke A, Fleckenstein P, Moises W, Beckmann H. [Studies of the reliability and validity of the German version of the Montgomery-Asberg Depression Rating Scale (MADRS)]. Schweiz Arch Neurol Psychiatr (1985). 1988;139(2):51-65.

2 Paulus W, Classen J, Cohen LG, Large CH, Di Lazzaro V, Nitsche M, et al. State of the art: Pharmacologic effects on cortical excitability measures tested by transcranial magnetic stimulation. Brain Stimul. 2008;1(3):151-63.

3 Goldman-Rakic PS. Cellular basis of working memory. Neuron. 1995;14(3):477-85.

4 Rossini PM, Pasqualetti P, Pozzilli C, Grasso MG, Millefiorini E, Graceffa A, et al. Fatigue in progressive multiple sclerosis: results of a randomized, double-blind, placebo-controlled, crossover trial of oral 4-aminopyridine. Mult Scler. 2001;7(6):354-8.

5 Smith A. Symbol Digit Modalities Test. Western Psychological Services: Portland; 1991.

6 Hossiep R, Turck D, Hasella M. BOMAT - advanced - short version: Bochumer Matrizentest*.* Hogrefe: Göttingen; 2001.

7 von Aster M, Neubauer A, Horn R. Wechsler Intelligenztest für Erwachsene (WIE). Deutschsprachige Bearbeitung und Adaptation des WAIS-III von David Wechsler. Harcourt Test Services: Frankfurt; 2006.

8 Shapiro SS, Wilk MB. An Analysis of Variance Test for Normality (Complete Samples). Biometrika. 1965;52(3/4).
